# Plant Volatile Organic Compounds Evolution: Transcriptional Regulation, Epigenetics and Polyploidy

**DOI:** 10.3390/ijms21238956

**Published:** 2020-11-25

**Authors:** Jesús Picazo-Aragonés, Anass Terrab, Francisco Balao

**Affiliations:** Departamento de Biología Vegetal y Ecología, Facultad de Biología, Universidad de Sevilla, Apdo. 1095, E-41080 Sevilla, Spain; anass@us.es (A.T.); fbalao@us.es (F.B.)

**Keywords:** volatile organic compounds, epigenetics, plant evolution, transcriptional regulation, polyploidy

## Abstract

Volatile organic compounds (VOCs) are emitted by plants as a consequence of their interaction with biotic and abiotic factors, and have a very important role in plant evolution. Floral VOCs are often involved in defense and pollinator attraction. These interactions often change rapidly over time, so a quick response to those changes is required. Epigenetic factors, such as DNA methylation and histone modification, which regulate both genes and transcription factors, might trigger adaptive responses to these evolutionary pressures as well as regulating the rhythmic emission of VOCs through circadian clock regulation. In addition, transgenerational epigenetic effects and whole genome polyploidy could modify the generation of VOCs’ profiles of offspring, contributing to long-term evolutionary shifts. In this article, we review the available knowledge about the mechanisms that may act as epigenetic regulators of the main VOC biosynthetic pathways, and their importance in plant evolution.

## 1. Plant Volatile Organic Compounds in the Epigenetic Era

Plants synthesize an amazing diversity of secondary metabolites, which have been selected throughout their evolutionary history as a response to specific needs [1,2]. Secondary metabolites are distinguished from primary metabolites (such as nucleic acids, amino acids, carbohydrates, etc.) in that they are specialized metabolites which have a wide range of functions, mediating interaction with both biotic and abiotic environments [3,4]. Volatile organic compounds (VOCs) are one of the most important secondary metabolites produced by plants. These lipophilic compounds have a low molecular weight and high vapor pressures at ambient temperature. More than 1700 VOCs have been identified in different species from both angiosperms and gymnosperms, including a total of 90 families and 38 orders [5]. They are fundamentally released from flowers, but also fruits, leaves, stems and even roots [6]. VOCs define the chemical landscape of numerous ecosystems by taking part in intra- and interspecific interactions [7,8,9], being highly context dependent, and functioning in direct and indirect ways from the landscape to the intrafloral scale [10]. Plant VOCs have effects on plant–pollinator, plant–herbivore, plant–plant and other interactions and, consequently, on fitness [11]. Among all these functions, the most important and understood one is the attraction of pollinators, ensuring the plant’s reproductive success [9,12]. Floral scent promotes plant–pollinator specialization, as well as outcrossing and reproductive isolation through floral constancy [10,13]. Thus, such a sexual signal is subject to high selective pressure, being fundamental for plants’ evolution and adaptation to the environment [11].

Nevertheless, VOCs are also crucial in plant defense against herbivores and protection from pathogens [8,14]. VOCs can act as repellents, being constitutively emitted [10,15] or showing an increased emission during herbivory attack [16]. In addition, specific VOCs can perform as indirect defenses, which can drastically reduce the number of herbivores, especially insects and larvae, by attracting parasitoids and predators [7]. Furthermore, bacteria-specific VOCs are emitted by plants with a diagnostic purpose, triggering defense signaling pathways and acting as direct inhibitors of bacterial growth, therefore making plants more resistant to pathogen invasion [17].

Although pollinator attraction and plant defense may be the most important functions of VOCs, they play many other roles in the interaction of plants with their environment. VOCs are essential in plan-to-plant signaling, allowing other plants to respond to herbivores and activate defenses before being attacked [8,18] They also work as allelochemicals and neighbor detection signals, taking part in competition between plants and also having an effect on the availability of environmental factors such as light, nutrients and water [19]. Moreover, plant VOCs are also involved in other less studied interactions, like symbiotic relationships with microorganisms and fungi [6] or attraction of seed dispersers [20]. In addition, abiotic stress factors, such as temperature and high light intensity, also stimulate VOC biosynthesis and emission [21,22]. Water, salt and oxidative stresses may increase VOC emissions, but their effects are not consistent throughout the literature [23]. VOC emission, especially that of terpenes, has been proven to mitigate these stresses, allowing plants to recover rapidly from high temperature exposure [24] or alleviating oxidative stress [25] and consequently increasing plant fitness.

To fulfill all these functions, plant VOCs are biosynthesized through entangled pathways which need complex regulation. VOCs are differentially—both temporally and spatially—synthesized throughout the development of the plant, due to differences in gene expression in distinct cell types [26]. This complexity is also illustrated by the high number of genes encoding enzymes from secondary metabolism (VOCs included), around 15%–25% out of 20,000–60,000 genes in plant genomes [1]. Over the last few years, recent progress in omics technology has led to a better understanding of plant VOC biosynthesis and the isolation of genes encoding candidate enzymes responsible for their regulation [27]. Nonetheless, the role of epigenetics in VOC biosynthesis and regulation has been neglected. Epigenetic regulation produces changes in gene function that can be mitotically and/or meiotically heritable and which do not entail a DNA sequence polymorphism [28]. The epigenetic signaling pathways altering the patterns of gene regulation and expression are cytosine DNA methylation (associated with both genes and transposable elements) and the post-transcriptional modification of histone proteins, which entails remodeling of chromatin structure [26]. Methylation of the 5′-position of cytosine residues is a reversible covalent modification of DNA, resulting in the production of 5-methyl-cytosine. Methylation changes the biophysical characteristics of DNA, causing the inhibition of DNA recognition by some proteins and allowing others to recognize it, which leads to the silencing of gene expression [29]. These proteins are known as methyl-binding proteins, transcriptional repressors that act through several mechanisms, such as the recruitment of corepressors and histone deacetylases, causing chromatin remodeling [30].

Additionally, RNA-based epigenetic mechanisms can also modify chromatin and silence transcription but are less understood. Non-coding RNAs have been known for a long time for their infrastructural role [31,32]. Furthermore, small (smRNA) and long non-coding RNAs (lncRNA) have recently emerged as key regulators of gene expression at the transcription level, RNA processing and translation [33,34]. Small RNAs can modify chromatin and silence transcription through the action of histone and DNA methyltransferases, recruited by guiding Argonaute-containing complexes to complementary nascent coding RNA or non-coding RNA scaffolds. They also act as a component of self-reinforcing positive feedback loops with an amplification component and participate in the epigenetic inheritance of histones and DNA methylation patterns. Moreover, lncRNA scaffolds recruit polycomb-group proteins and other chromatin-modifying complexes independently of smRNAs, but this mechanism remains poorly understood [35].

In this context, epigenetic mechanisms may be essential for plants due to their reversible nature and the huge influence of environmental conditions [26,31,32]. Epigenetic factors have recently emerged as relevant modulators of rapid plant responses to environment, enabling plants to face recurring stress events more efficiently and also preparing offspring for future adversities [36,37].

Therefore, it seems reasonable that epigenetics would play a main role in the regulation of VOC emissions in plants. In this respect, polyploidy (whole genome duplication) could act as an important driver of VOC evolution. Polyploidy in plants has multiple effects at different scales, from the molecular (including the epigenetic landscape) to the phenotypic level, owing to advantageous evolutionary success [38]. Moreover, the persistence of epigenetic information through meiosis (i.e., transgenerational epigenetic inheritance) could also contribute to evolution of the temporal dynamic of VOCs and the plant’s biotic interactions [39]. To gain insights about the evolution of floral scent profiles and their temporal regulation, we review here the current knowledge about the transcriptional regulation of the different families of VOCs and the influence of epigenetic mechanisms and polyploidy on their biosynthesis and emission.

## 2. Biosynthesis and Epigenetic Regulation of VOCs

Biosynthesis of plant volatiles, as well as other secondary metabolites, depends on the availability of carbon, nitrogen and sulfur, as well as energy, exposing the connection between primary and secondary metabolism. From only a few of these primary metabolic pathways, an extensive array of different routes branches off [4]. However, despite the fact that there is a huge diversity of volatile organic compounds, most of the biosynthetic pathways are conserved across plant kingdoms [40,41]. Based on their biosynthetic origin, VOCs can be classified into three major groups: terpenoids, phenylpropanoids/benzenoids and fatty acid derivatives. Although the upstream enzymatic steps of the main biosynthetic pathways have been elucidated, knowledge about the last enzymatic steps and their regulation is scarce and is limited to model, crop and medicinal plants with high added value.

### 2.1. Genetic Roadmap to Terpenoid Biosynthesis and Regulation

**Figure 1:**
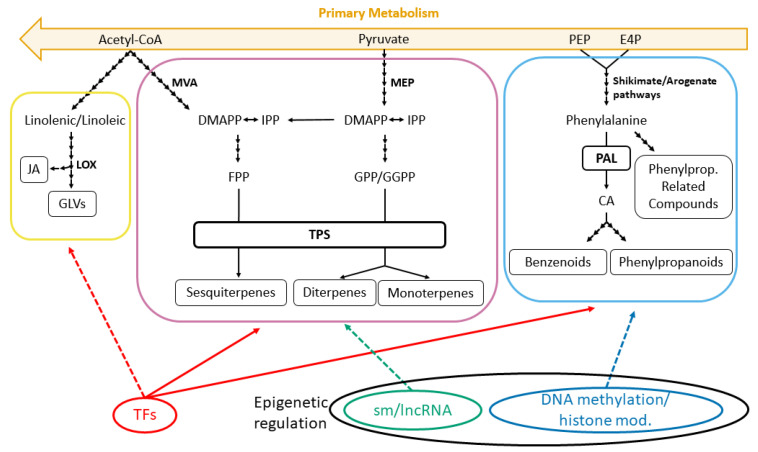
Overview of the main volatile organic compounds’ biosynthetic pathways: terpenoids (pink), phenylpropanoids/benzenoids (blue) and fatty acid derivatives (yellow). Volatile organic compounds (VOCs) are secondary metabolites of which the precursors are products of primary metabolism. These are transformed into volatile terpenes (monoterpenes, diterpenes and sesquiterpenes) by the mevalonic acid (MVA) and methyleruthirol phosphate (MEP) pathways. Phenylpropanoid/benzenoid biosynthesis follows the shikimate and arogenate pathways. Meanwhile, fatty acid derivatives such as jasmonate (JA) and green leaf volatiles (GLVs) come from the lipoxygenase (LOX) pathway. Volatile terpenoids and phenylpropanoids are transcriptionally controlled by transcription factors (continuous lines) and may be epigenetically controlled by small and long non-conding RNA, as well as by DNA methylation and histone modification (dashed lines). Volatile fatty acid derivatives may be regulated by transcription factors (TFs) and epigenetic mechanisms.

Terpenoids are the largest family of VOCs and encompass over 550 compounds [42]. These are derived from two common five-carbon (C_5_) precursors, isopentenyl diphosphate (IPP) and its allylic isomer, dimethylallyl diphosphate (DMAPP) [43]. In plants, these C_5_-isoprene precursors are synthesized from two independent and compartmentally separated pathways: the mevalonic acid (MVA) pathway and the methylerythritol phosphate (MEP) pathway. The MEP pathway occurs exclusively in plastids [44], providing precursors for volatile hemiterpenes (C_5_), monoterpenes (C_10_) and diterpenes (C_20_), whereas the MVA pathway is distributed among the cytosol, endoplasmic reticulum and peroxisomes [45,46], producing precursors for volatile sesquiterpenes (C_15_). Despite being compartmentally separated, MVA and MEP pathways are connected by metabolic crosstalk [47], allowing the MEP pathway, often with a higher carbon flux, to support the biosynthesis of cytosolically formed terpenoids [47,48,49]. The MVA and MEP pathways are well characterized and genes are conserved across plant species, producing prenyl diphosphate precursors (farnesyl diphosphate (PP) and geranyl diphosphate (GPP)), which are substrates of terpene synthases/cyclases (TPSs) [4,42,50]. At the final steps of the pathways (Figure 1), TPSs are responsible for the tremendous diversity of volatile terpenoids in plants [51]. Almost half of the known TPSs can synthesize multiple products from a single substrate [51], and many of them accept more than one prenyl diphosphate precursor [52,53], which increases the diversity of the produced terpenoids by directing bifunctional enzymes to different compartments with a wide range of available substrates [49,54].

The *TPS* gene family is a mid-size family that includes approximately 20 to 150 genes, identified in a variety of plant species. TPSs are present in all plant genomes and they can be phylogenetically classified into seven subfamilies (TPS-a–TPS-h) [55]. Most of them have been isolated from vegetative tissues and fruits to investigate their presumably defensive function [56], but their role in floral attraction might be equally important from the perspective of plant evolution and speciation [57]. In contrast to the vast information about terpenoid biosynthesis-related enzyme-encoding genes, the regulatory network controlling the expression of TPS genes at the transcriptional level remains unclear. However, at least six transcription factor (TF) families are implied in the regulation of terpenoid biosynthesis (Figure 1): AP2/ERF, bHLH, bZIP, ARF, MYB and WRKY [58,59,60]. In *Arabidopsis*, the TFs AtMYB21 and AtMYC2 regulate the expression of two sesquiterpene synthase genes, *TPS11* and *TPS21*, via the gibberellic and jasmonic acid (JA) signaling pathways [58,61]. The role of these MYB TFs have been also shown in *Freesia* × *hybrid*, which has eight TPS genes responsible for the spatiotemporal release of VOCs [56]. Alternatively, in gymnosperms (i.e., *Pinus taeda*), *PtMYB14* regulates the terpenoid MVA pathway [62]. It was recently shown that MYB TFs interact with other bHLH TFs to form a MYB–bHLH complex, participating in the activation of sesquiterpene biosynthesis [63,64]. In addition, AtARF6 and AtARF also regulate the synthesis of sesquiterpenes by binding to *TPS11* and *TPS21* promoters [58]. Finally, the defensive terpene emission in *Nicotiana attenuata* seems to be controlled by the TFs NaWRKY3 and NaWRKY6 [65].

Furthermore, the temporal emission of TPSs is controlled by the circadian clock [66], which is under epigenetic control, as explained in further sections. In addition, microRNAs (miRNAs) and long noncoding RNAs (lncRNAs) play a key role in the terpenoid regulatory network (Figure 1). These non-protein-coding transcripts target terpene biosynthesis pathway genes in rose-scented geranium species (*Pelargonium* spp.) [67]. In addition, miRNAs and lncRNAs regulate terpene trilactone (TTL) biosynthesis by targeting structural genes and TFs in *Ginkgo biloba* [68].

### 2.2. Regulation of Transcription Factors Affects Phenylpropanoid/Benzenoid Biosynthesis

Phenylpropanoids and benzenoids constitute the second largest class of plant VOCs [5] and are derived from the aromatic amino acid phenylalanine (Phe), which is synthesized via two alternative branched pathways that connect central carbon metabolism to Phe—the shikimate and the arogenate pathways [69,70]. This VOC class can be subdivided into three different subclasses depending on the structure of their carbon skeleton—phenylpropanoids (with a C_6_-C_3_ backbone), benzenoids (C_6_-C_1_) and phenylpropanoid-related compounds (C_6_-C_2_) [42] (Figure 1).

The first committed step in the biosynthesis of the majority of phenylpropanoid and benzenoid compounds is catalyzed by the well-known and widely distributed enzyme L-phenylalanine ammonia-lyase (PAL), which catalyzes the deamination of Phe to produce trans-cinnamic acid (CA) [70]. Benzenoid formation from CA can occur via a β-oxidative pathway, a non- β-oxidative pathway or a combination of both, which are well characterized [71,72]. Recently, the BAHD superfamily of acetyltransferases [73] and the SABATH family of methyltransferases [74] have been found to contribute to the final biosynthetic steps of volatile benzenoids. The formation of volatile phenylpropanoids shares initial biosynthetic steps with lignin biosynthesis up to phenylpropenol (monolignol precursor), which is then converted into coniferyl alcohol [75,76]. A coniferyl alcohol acetyltransferase from the BAHD superfamily forms coniferyl acetate [75], which is the precursor of the common phenylpropanoids eugenol and isoeugenol [76,77].

Furthermore, volatile phenylpropanoid-related compounds originate directly from Phe, not via CA [78,79]. Only genes and enzymes responsible for the biosynthesis of phenylacetaldehyde and 2-phenylethanol have been isolated and characterized. Phenylacetaldehyde production by phenylacetaldehyde synthasis depends on biosynthetic pathways that differ between organisms [78,79,80,81,82]. Further conversion of phenylacetaldehyde to 2-phenylethanol is catalyzed by a phenylacetaldehyde reductase, as shown in roses [83,84].

Transcriptional regulation of phenylpropanoid/benzenoid biosynthesis (Figure 1), and hence scent emission, is controlled by TFs [85]. ODORANT1 (ODO1) and EMISSION OF BENZENOIDS II (EBOII) are R2R3-type MYB TFs responsible of the broad control of phenylpropanoids/benzenoids in *Petunia* × *hybrida* by regulation of the shikimate pathway, as well as the entry points into both the Phe and phenylpropanoid branchways [86]. Suppression of *ODO1* and *EBOII* expression in *P.* × *hybrida* significantly decreased the transcript level of many phenylpropanoid/benzenoid genes (including *DAHPS*, *EPSPS*, *PAL*, *CM* and *SAMS*) and therefore decreased the emission of most floral volatiles [86,87]. EBOII also activates the *ODO1* promoter, favoring the emission of volatile phenylpropanoids/benzenoids [88]. EMISSION OF BENZENOIDS I (EBOI) is a flower-specific R2R3-type TF, closely related to *EBOII* and upregulated by the EBOII TF, that directly activates *ODO1* and numerous scent-related genes [86]. In contrast to ODO1, EBOII and EBOI, the MYB4 TF is a specific repressor of the cinnamate-4-hydroxylase from the phenylpropanoid pathway, consequently controlling the flux towards phenylpropanoid volatile compounds in petunias [89]. Recently, R2R3-MYB TFs were found to regulate flavonol biosynthesis through targeting flavonol synthase (FLS) genes in *Freesia* × *hybrida* [90]. *FLS* genes are responsible for the spatio-temporal biosynthesis of floral flavonols and are regulated by the well-known SG7 MYB protein family [85,91], but MYB21 was also found to regulate *FLS* [90]. The MYB21 TF has been considered a terpenoid biosynthesis regulator, so there might exist a connection between terpenoids and flavonoid biosynthesis, but it is still unclear.

Little is known about the epigenetic regulation of phenylpropanoids/benzenoids. Methylation and histone modification have been shown to epigenetically regulate different non-volatile phenylpropanoid biosynthesis pathway genes (Figure 1). In ash trees (*Fraxinus excelsior*), the enzyme cinnamoyl-CoA reductase 2 is encoded by a gene that showed different methylation patterns, causing differences in the expression of phenylpropanoid genes [92]. DNA/histone methylation also alters transcription levels of phenylpropanoid pathway genes involved in anthocyanin biosynthesis in potatoes [93], and other defensive and structural phenylpropanoids such as lignin in maize [94], which share some steps with the volatile phenylpropanoid biosynthesis. Since methylation, together with MYB transcription factors, has been shown to globally control phenylpropanoid/benzenoid biosynthesis genes involved in defense and physiological functions, it could also regulate floral phenylpropanoid/benzenoid biosynthesis and emission. In fact, *ODO1* presents a conserved cis-regulatory element in its promoter related to circadian control of emission [95], which seems epigenetically controlled through chromatin remodeling (see below). Provided that the results could be extrapolated to the volatile phenylpropanoids, epigenetics may play an important role in its biosynthesis and emission, but further investigation is needed.

### 2.3. Crosstalk between Fatty Acid Derivatives, Biosynthesis and Epigenetics

Fatty acid derivatives such as (*Z*)-3-hexenol, 2-ketones and methyl jasmonate constitute the third major class of VOCs, which derive from C_18_ unsaturated fatty acids, linoleic acids or linolenic acids. Biosynthesis of these compounds depends on the plastidic pool of acetyl-CoA generated from pyruvate (Pyr), the last product of glycolysis. The lipoxygenase (LOX) pathway leads to the formation of several VOC intermediates from two different branches. The allene oxide synthase (AOS) branch leads to the formation of jasmonic acid (JA), which is converted to methyl jasmonate by JA carboxyl methyl transferase [96]. In contrast to the AOS branch, the hydroperoxide lyase branch converts both 9- and 13-hydroperoxy intermediates into C_6_ and C_9_ aldehydes, which are often substrates for alcohol dehydrogenases, giving rise to volatile alcohols and their esters [97]. These saturated and unsaturated C_6_/C_9_ aldehydes and alcohols, known as green leaf volatiles (GLVs), are usually synthesized in green plant organs of plants in response to wounding, and they also provide aroma to fruits and vegetables. However, these fatty acid derivatives are also important constituents of the floral volatile bouquet of several plant species (e.g., carnation, oil-secreting *Lysimachia* and wild snapdragon) [98,99,100].

The biosynthetic pathway of the volatiles derived from fatty acids is the least studied of all VOCs (Figure 1), but even less is known about its transcriptional regulation. Studies have usually focused on the defensive role of fatty acid derivatives and most of the information about their transcriptional regulation refers to jasmonate (JA) and its key role in the biosynthesis of other secondary metabolites [101,102]. Many TFs, such as MYC (MYC2,3 and 4), MYB, EIN3, EIL, ERF, GAI, RGA and RGL1, may take part in the regulation of JA biosynthesis and coordination with other secondary metabolites [103]. For example, JA is part of the volatile sesquiterpene’s regulatory transcriptional network and is controlled by ARF6 and ARF8 TFs [61].

Recent studies have demonstrated a correlation between acetyl-CoA (a fatty acid volatile precursor), β-oxidation and the redox state of plant cells, which affect the global epigenetic pattern [104,105]. Acetyl-CoA is produced by β-oxidation in plants and plays critical roles in development and secondary metabolite biosynthesis. Defects in the β-oxidation pathway may alter cells’ redox state, affecting DNA methylation and histone acetylation in the nucleus [104,106]. As biosynthesis of fatty acid derivative volatiles is affected by the redox state of cells (and vice versa) [107], it is conceivable that this represents an epigenetic component in its regulation. In this respect, other fatty acid derivatives not related to floral volatiles have been shown to be epigenetically controlled. In *Arabidopsis thaliana* seeds, oil composition is altered by histone acetyltransferases which cause changes in the expression of genes from the fatty acid biosynthetic pathway [108]. Furthermore, in *Triticum aestivum*, TFs binding to the enoyl-CoA reductase gene promoter (a main component in wax biosynthesis) recruit histone acetyltransferases that alter their expression [109]. Although these biosynthetic pathways are not closely related to floral VOC biosynthesis, there may exist similar mechanisms regulating fatty acid derivatives, so further investigation is needed in this area.

## 3. The Temporal Pattern of Emissions and Its Epigenetic Regulation

Regulation of the rhythmic release of VOCs under circadian control has been a topic of investigation for the past several years. The release of floral scent at a specific time of the day is thought to be evolutionarily beneficial in order to maximize resource efficiency, attracting only truly effective pollinators and also avoiding the attraction of predators, which reduce herbivory [110,111]. These temporal patterns are accompanied by oscillations in the expression of genes in the VOC biosynthetic pathways [110,112]. Temporal patterns of phenylpropanoid/benzenoid emission have been studied in multiple plant genera, such as *Petunia, Nicotiana* and *Antirrhinum* [89,113,114,115]. In addition, other studies have also reported a rhythmic emission of terpenes, but little is known about the circadian regulation of volatile fatty acid derivatives [111,116].

**Figure 2:**
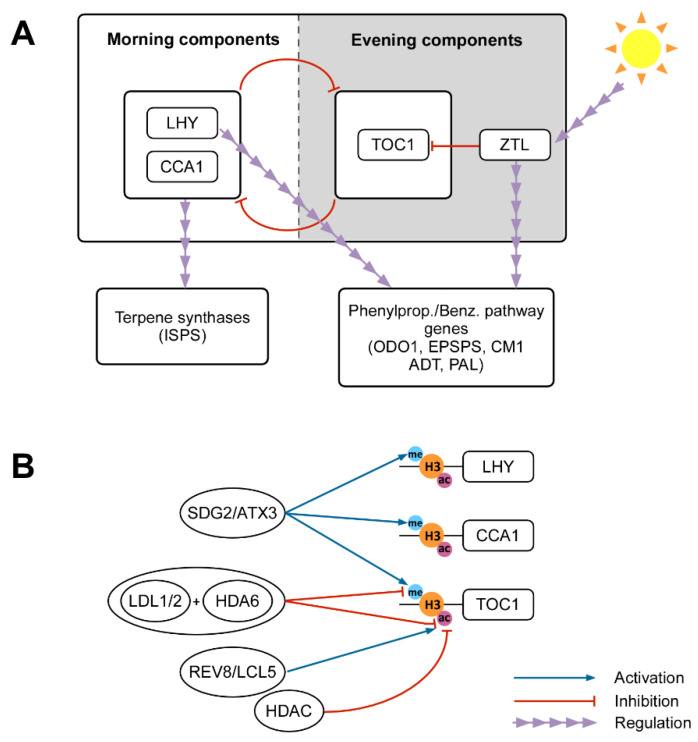
Circadian clock regulation of VOC emission and its epigenetic regulation. (**A**) The main oscillator of the circadian clock is formed of the morning components *CIRCADIAN CLOCK ASSOCIATED 1 (CCA1)* and *LATE ELONGATED HYPOCOTYL (LHY)* and the evening components *TIMING OF CAB EXPRESSSION 1 (TOC1)* and *ZEITLUPE (ZTL)*. The morning and evening components regulate each other in a negative feedback loop. Light regulates *ZTL* expression, which ubiquitinates TOC1 for degradation. These clock genes control the daily expression of terpene synthases and phenylpropanoid/benzenoid pathway genes. (**B**) Methylation and acetylation of the H3 histone of the main clock genes causes chromatin remodeling and subsequent changes in these genes’ expression, affecting the biosynthesis and emission of VOCs. Histone deacetylases (HDACs), induced by TOC1 and LHY, cause H3 deacetylation in the *TOC1* promoter, repressing it. Meanwhile, the REVEILLE 8/LHY-CCA1-LIKE 5 (RVE8/LCL5) TF has an opposite role, promoting hyperacetylation of H3 in the *TOC1* gene. Di- and trimethylation of H3 histone also alters the expression of *CCA1*, *TOC1* and *LHY*. In *Arabidopsis* SET DOMAIN GROUP 2/ARABIDOPSIS TRITHORAX RELATED 3 (SDG2/ATXR3) and LDL1-HDA6 alter methylation and acetylation patterns of these circadian genes.

Circadian rhythmic emission of volatiles is regulated by the expression of key genes, which may also determine the rhythmicity of downstream volatile emissions, modifying the expression of genes at the final steps of biosynthetic pathways. Genes implied in VOC biosynthesis can be temporally regulated by transcription factors (TF) coded by clock genes [4,110], which may act as regulators of the rhythmic emission of volatiles [111]. The main components of the circadian clock have been elucidated in *Arabidopsis thaliana* [117] but they are highly conserved across many plant species [118]. The *CIRCADIAN CLOCK ASSOCIATED 1 (CCA1)* and *LATE ELONGATED HYPOCOTYL (LHY)*, known as morning components, along with the response regulator CCT-domain protein *TIMING OF CAB EXPRESSSION 1 (TOC1)* and *ZEITLUPE (ZTL)*, known as evening components, form the main oscillator at the core of the plant circadian clock, based on transcriptional/translational negative feedback loops involving these clock genes (Figure 2A). *CCA1* and *LHY* inhibit the expression of *TOC1*; the TOC1 protein also represses *CCA1/LHY*; and ZTL ubiquitinates TOC1 for degradation as a response to blue light [119,120]. The daily oscillation of light and temperature are the main exogenous cues for the plant’s circadian clock [121]. In the gray poplar (*Populus* × *canescens*), diurnal variation in terpene emission is associated with oscillations in the expression of genes encoding for isoprene synthases (ISPS), which are controlled by the presence of circadian regulatory elements (CCA1/LHY) in the promoter sequence of *ISPS* [66]. Furthermore, the clock gene *LHY* in *Petunia* × *hybrida* and tobacco (*Nicotiana attenuata*) controls the daily expression pattern of many phenylpropanoid/benzenoid genes and TFs (*ODO1*, *EPSPS*, *CM1*, *ADT*, *PAL*, etc.) and hence the production of these floral volatiles [111]. Although the expression of *LHY* leads to a decrease in the expression of many phenylpropanoid/benzenoid genes and the loss of emission, reducing its constitutive expression has an opposite effect [114,122]. In addition, *ZTL* repression in tobacco (and its ortholog in petunia, *Petunia CHANEL* (*PhCHL*)) leads to a decrease in floral scent emission [122].

Recent studies have demonstrated the epigenetic regulation of circadian clock components [123,124,125]. Chromatin reshaping by histone acetylation, methylation and phosphorylation (Figure 2B) may create a flexible loop of clock gene regulation [125]. The CCA1 morning factor binds to the core oscillator evening gene *TOC1* promoter, inducing deacetylation of the histone H3 by histone deacetylases (HDACs) that causes chromatin compaction and subsequent *TOC1* repression at dawn [121,124]. In addition, the REVEILLE 8/LHY-CCA1-LIKE 5 (RVE8/LCL5) TF, which also has a morning peak like CCA1 and LHY, promotes hyperacetylation of H3 in the *TOC1* promoter, activating *TOC1* expression and having an opposite role in its regulation. This may facilitate *TOC1* rising phases and provide a fine-tuning mechanism for precisely shaping the rhythmic expression of *TOC1* [126].

Together with H3 acetylation, methylation (di- and trimethylation) of the histone H3K4 (H3K4me3 and H3K4me2) constitutes the main histone marks in clock gene regulation, altering the expression period of *CCA1* and *TOC1*. In *Arabidopsis,* the H3K4 methyltransferase SET DOMAIN GROUP 2/ARABIDOPSIS TRITHORAX RELATED 3 (SDG2/ATXR3) contributes to H3K4me3 accumulation and the expression of oscillatory genes [121,123]. Furthermore, another histone mark regulating clock gene expression is H3K36me2, of which the accumulation at *TOC1*, *CCA1* and *LHY* promoters is associated with their transcriptional repression [127]. At the same time, LYSIN-SPECIFIC DEMETHYLASE 1 (LSD1)-LIKE histone demethylases LDL1 and LDL2 interact with *CCA1* and *LHY* to repress *TOC1* expression. LDL1 and LDL2 interact with histone deacetylase HDA6, and the LDL1-HDA6 complex binds to the promoter of *TOC1* and represses its expression by deacetylation and demethylation [128].

In plants, the circadian clock core loop is self-regulated by the interaction of its clock genes. Epigenetic modifications are part of this complicated regulatory system, in which morning components repress the evening ones and vice versa, controlling the different clock gene expressions by means of histone modifications and chromatin reshaping, and thus fine-tuning the changes between day and night phases. The proposed epigenetic model and its precise regulation of the circadian clock would impact VOC emission at multiple levels in the short- and long-term (i.e., during the plant’s lifespan and subsequent generations), influencing plant fitness and natural selection [129].

## 4. Evolution of VOCs: The Role of Polyploidy and Transgenerational Memory

Floral scent is considered an easily evolved trait, as VOCs are usually acquired and lost in related species [130,131]. Scent compounds would have mostly evolved due to gene duplication and divergence–convergence [1,2]. The large gene families, terpene synthases and O-methyl transferases, among others, would have evolved following this mechanism [1,55]. In addition, these modifications can also occur without the mediation of duplication events on single genes, changing the VOC emission throughout the evolutionary history [132]. Structural mutations modifying the enzyme catalytic sites can also cause the gaining of new functions (or the loss thereof) and hence cause changes in VOC emission. However, more interestingly, scent changes (qualitatively and quantitatively) can arise due to gene expression changes. Gene silencing or activation cannot only affect an individual VOC emission, but it can also have a cascade effect on the complete pathway [46,133]. New VOC emissions can straightforwardly arise from exogenous gene expression in new tissues. For example, in *Clarkia breweri*, linalool emission (its most abundant floral VOC) most likely evolved from trace emissions in stigma tissue in the ancestor *C. concinna* [134]. Such VOC changes could arise as a consequence of epigenetic regulation of genes, creating advantageous or disadvantageous phenotypes that are subject to natural selection. If new VOCs confer a selective advantage for the plant, the epigenetic change and the VOC production would be retained and inherited [135]. The transgenerational inheritance of floral and leaf VOCs has been recently found in *Brassica rapa* [136], and this could be epigenetically mediated [39]. Therefore, epigenetic change would propose a flexible evolutionary mechanism and help to explain the continuous gain and loss of specific VOCs in related plant lineages. Epigenetic variations would assist plants in surfing on an adaptative landscape in which abiotic and biotic interactions usually involve trade-offs (e.g., herbivory and pollinator attraction) [137,138].

Furthermore, all angiosperm lineages show vestiges of past rounds of whole genome duplication (WGD), highlighting the role of polyploidy as a major driving force in plant evolution [139,140,141]. Polyploidy has multiple effects at different scales, from the molecular to the phenotypic level, owing to the advantageous evolutionary success [38]. Patently, WGD (and the consequent gene duplication) is an important molecular evolutionary mechanism driving the large diversity of plant VOCs. In addition, following WGD, polyploids suffer changes in genomic structure and epigenetic remodeling [142,143]. Polyploidy usually causes an altered pattern of DNA methylation in genes, promoters and transposable elements [144,145]. These changes are enhanced in allopolyploids (WGD associated with species hybridization) because of “genomic shock”. In this case, this methylation repatterning is related to parental dominance (i.e., the higher expression of one parental homeolog) and fractionalization [38]. In addition, changes in smRNAs and chromatin have also been observed after WGD [146,147,148]. In this way, autopolyploidy (WGD without hybridization) has been recently considered as an epigenetic macromutation affecting chromatin compaction and altering contacts among chromosomes (i.e., 4D nucleome) as a side effect of the increased nucleus size [149]. This altered epigenetic landscape may promote divergent gene expression by regulating mobile element activity and silencing redundant genes [144,150]. In addition, these epigenetic alterations could create many opportunities for natural selection to act, enhancing polyploid establishment [151].

Concurrently to the epigenetic changes, WGD also exerts a cornucopia of effects on the plant phenotype, including numerous floral traits [16]. Changes in scent pattern have been shown in some orchid polyploids [152,153]. Such changes in floral traits could impact pollinator attraction and lead to differentiation in the pollinator spectrum [132,154], causing the isolation of diploids and polyploids and facilitating polyploid establishment. Although gene duplication and later neofunctionalization [155,156] can promote such scent changes, rapid scent shifts could be presumably attributed to epigenetic remodeling following WGD [39,136]. Therefore, the role of the epigenetic change associated with polyploidy on scent biosynthesis and emission (and its evolutionary contribution) deserves further investigation.

In this respect, the carnation complex of *Dianthus broteri* is an appropriate study system to investigate the interplay between epigenetics and polyploidy on the evolutionary change of floral volatiles. This Iberian endemic complex represents the most extensive polyploid series of the *Dianthus* genus, with diploid (2n = 30), tetraploid (2n = 60), hexaploid (2n = 90) and dodecaploid (2n = 180) individuals [157]. Molecular and genome size studies have demonstrated that it is an autopolyploid complex [158], in which the different cytotypes form independent and monophyletic lineages and show disjunct geographic distributions with monocytotypic populations and different ecological niches within an aridity gradient, with the higher cytotypes (6× and 12×) being those that inhabit more restricted and extreme habitats [159,160]. It is also remarkable that, in this complex, polyploidy leads to an increased degree of DNA methylation [161] and of epigenetic variability, which may be crucial to phenotypic divergence (i.e., changes in VOCs), adaptation and shifts in biotic interactions [37,162,163]. Previous studies [16,164] have shown phenotypic differences in floral traits (floral size, distance to access the nectar and scent) between *D. broteri* cytotypes that may promote changes in pollinator patterns. The highest ploidy level (12×, *D. inoxianus*) involves a highly specialized pollination by a single pollinator, *Hyles livornica*. The floral scent of this cytotype is dominated by sesquiterpenoids and fatty acid derivatives (2-ketones), which shown a circadian emission [16]. The 2-ketones elicit responses in the antennae of the single pollinator, but also have a repellent/insecticide activity [165]. They show an explosive increase in production, triggered by experimental damage to *D. inoxianus* flowers, which indicates that 2-ketones may be acting like a floral filter of the alleged pollinator spectrum, leading to pollinator specialization [16]. Supporting this “filtering hypothesis”, these ketones appear in low quantities at diploid and tetraploid levels, which show a wider pollinator spectrum [166]. In addition, dominant aromatic compound changes (e.g., the disappearance of β-ocimene in 4× and 12× and the dominance of β-caryophyllene in 2× and 4×) suggest that shifts in the expression of scent-related genes may be promoting greater specialization in higher polyploids. We suggest a combination of epigenomics, biochemical and pollination biology methods to investigate whether epigenetics is involved in polyploid evolution by means of floral VOCs and pollinator shifts.

## 5. Final Remarks

Elucidating the pathways responsible for VOC biosynthesis and the mechanisms behind its regulation are crucial in order to understand the role of volatile organic compounds in plant adaptation and evolution. Although terpenoid and phenylpropanoid/benzenoid biosynthetic pathways are better understood, much less is known about fatty acid derivatives. Transcription factors are key regulators of terpenoid and phenylpropanoid/benzenoid biosynthesis, which are also controlled by the circadian clock. In this sense, the circadian clock is subject to epigenetic regulation, altering the daily emission of VOCs. In addition, other epigenetic modifications such as DNA/histone modification and lncRNAs can modify the expression of genes and TFs in phenylpropanoid and terpenoid biosynthetic pathways. Additionally, the relationship between plant polyploidy and epigenetics is essential in explaining the massive diversity of VOCs and their importance in plant evolution. However, most of our knowledge on the epigenetic regulation of VOC biosynthesis and emission is conjectural and based on studies of non-volatile compounds. We propose *Dianthus broteri* as suitable system to study the outlined research gaps about the role of epigenetics and polyploidy on the evolutionary change of floral volatiles.
